# Targeting Lipid Rafts as a Strategy Against Coronavirus

**DOI:** 10.3389/fcell.2020.618296

**Published:** 2021-02-04

**Authors:** Maurizio Sorice, Roberta Misasi, Gloria Riitano, Valeria Manganelli, Stefano Martellucci, Agostina Longo, Tina Garofalo, Vincenzo Mattei

**Affiliations:** ^1^Department of Experimental Medicine, “Sapienza” University, Rome, Italy; ^2^Biomedicine and Advanced Technologies Rieti Center, “Sabina Universitas”, Rieti, Italy

**Keywords:** coronavirus, SARS-CoV-2, lipid rafts, statins, cyclodextrins

## Abstract

Lipid rafts are functional membrane microdomains containing sphingolipids, including gangliosides, and cholesterol. These regions are characterized by highly ordered and tightly packed lipid molecules. Several studies revealed that lipid rafts are involved in life cycle of different viruses, including coronaviruses. Among these recently emerged the severe acute respiratory syndrome coronavirus-2 (SARS-CoV-2). The main receptor for SARS-CoV-2 is represented by the angiotensin-converting enzyme-2 (ACE-2), although it also binds to sialic acids linked to host cell surface gangliosides. A new type of ganglioside-binding domain within the N-terminal portion of the SARS-CoV-2 spike protein was identified. Lipid rafts provide a suitable platform able to concentrate ACE-2 receptor on host cell membranes where they may interact with the spike protein on viral envelope. This review is focused on selective targeting lipid rafts components as a strategy against coronavirus. Indeed, cholesterol-binding agents, including statins or methyl-β-cyclodextrin (MβCD), can affect cholesterol, causing disruption of lipid rafts, consequently impairing coronavirus adhesion and binding. Moreover, these compounds can block downstream key molecules in virus infectivity, reducing the levels of proinflammatory molecules [tumor necrosis factor alpha (TNF-α), interleukin (IL)-6], and/or affecting the autophagic process involved in both viral replication and clearance. Furthermore, cyclodextrins can assemble into complexes with various drugs to form host–guest inclusions and may be used as pharmaceutical excipients of antiviral compounds, such as lopinavir and remdesivir, by improving bioavailability and solubility. In conclusion, the role of lipid rafts-affecting drugs in the process of coronavirus entry into the host cells prompts to introduce a new potential task in the pharmacological approach against coronavirus.

## Coronaviruses

Coronaviridae family is morphologically characterized by a crown shape deriving from the presence on the envelope of a 20-nm long protein called “spike” (Cong and Ren, 2014; Tortorici and Veesler, 2019). It is possible to divide the coronaviridae family into four genera: α, β, γ, and δ coronaviruses. To date, there are 46 known coronaviruses species (ICT, 2019) that infect several hosts, including humans, mammals, birds, and other animals; in particular, when considering humans, they are infected mainly by both α- and β-coronaviruses (Geng et al., 2020). β-Coronaviruses can be further subdivided into five subgenes, namely, Embecovirus, Hibecovirus, Merbecovirus, Nobecovirus, and Sarbecovirus (ICT, 2019; Jeong-Min et al., 2020).

Coronavirus is an RNA virus with positive single-stranded RNA. Regarding the virus structure, this family is characterized by a lipid coating deriving from the host called “envelope” and a nucleocapsid in which the genetic material is contained. The envelope, in addition to the transmembrane glycoprotein named M and the envelope protein (E), contains the spike glycoprotein involved in the process of recognizing the host cell. Spike protein differs for point mutation in coronaviruses (Wang Q. et al., 2020; Yan et al., 2020). Moreover, the nucleocapsid is helical in shape and consists of a positive polarity polyadenylated RNA molecule equipped with CAP and associated with protein N.

The mechanisms of coronaviruses entry are complex and differ among coronavirus species and strains. Coronaviruses entry can occur by direct fusion at the cell surface after binding to the receptor or after internalization via endocytosis with fusion taking place in the endosomal compartment (Belouzard et al., 2012; Wȩdrowska et al., 2020). The main mechanism of coronaviruses entrance is based on spike protein that is the primary determinant of cell tropism. Spike is a class I transmembrane protein, synthesized as a precursor protein with a typical size ranging from 1,200 to 1,300 amino acids (Wang Q. et al., 2020). The fusion mechanism of viral membranes with host membranes is related to conformational changes of the spike protein (Belouzard et al., 2012; Wȩdrowska et al., 2020). In particular, several coronaviruses may enter directly from the cell surface, when receptor-bound viruses are treated with proteases activating S proteins. This process generates homotrimers on the virion surface triggering the early fusion pathway. Alternatively, coronavirus may be endocytosed within the endosome where the low pH activates cathepsin L, cleaving S2′ site, triggering the fusion pathway, and releasing the coronaviruses genome (Tang et al., 2020).

In November 2019, a new virus named SARS-CoV-2, belonging to the Coronaviridae family, appeared in Wuhan for the first time. In March 2020, the World Health Organization (WHO) declared pandemic the viral disease caused by this virus. SARS-CoV-2 virus has been isolated from nasopharyngeal and oropharyngeal samples from patients affected with a flu-like disease (Jeong-Min et al., 2020). To date, there are several hypotheses on the SARS-CoV-2 origins; the most accredited hypothesis by scientists regard the transmission from wild animals to humans. In fact, several wild animals serve as a reservoir for new coronaviruses; these include bats, pangolins, and others. In a recent work, Lam et al., by metagenomic sequencing, have identified some new coronaviruses isolated from the pangolin that show a high similarity with SARS-CoV-2 in the receptor binding domain (Lam et al., 2020).

Coronaviruses that infect humans are involved in acute respiratory diseases, including colds, pharyngitis, nasal congestion, as well as, in some cases, headache, cough, muscle pain, and fever.

The clinical courses of infected hosts may be vary, ranging from asymptomatic, mild symptoms, or severe symptoms and cause respiratory, enteric, hepatic, and neurological diseases (Monchatre-Leroy et al., 2017; Cui et al., 2019). At present, seven types of coronavirus are known as inducing infections in humans. In particular, the species HCoV-229E, HCoV-OC43, HCoV-NL63, and HCoV-HKU1 usually cause mild symptoms, whereas SARS-CoV-2, SARS-CoV, and Middle East respiratory syndrome coronavirus (MERS*-*CoV) are able to cause severe respiratory disease like pneumonia and death (de Wit et al., 2016; Corman et al., 2018; Walls et al., 2020).

Infected people can be asymptomatic or present a flu-like disease with an incubation period that can vary from 2 to 14 days during which the individual is able to transmit the virus. From current data, it has been estimated that in 3–15% cases, the virus can lead to a severe respiratory disease as pneumonia and cause death. The large majority of deaths is represented by elderly people over 70 years of age and with comorbidities.

The emergency caused by the SARS-CoV-2 infection in Wuhan has spread to many countries and forced the WHO to declare a pandemic in March 2020. The SARS-CoV-2 infection is currently underway and is exponentially developing especially in USA, Europe, South America, Russia, and India, recording more than 2,000,000 deaths.

## Lipid Rafts

Lipid rafts are highly dynamic structures that can play a key role in pathogens–cell interactions, including coronaviruses–host cell (Carotenuto et al., 2020; Fecchi et al., 2020).

Lipid rafts are functional membrane microdomains that contain sphingolipids and cholesterol. These regions are characterized by a highly ordered and tightly packed lipid molecules compared to the surrounding bilayer (Simons and Ikonen, 1997; Wang and Silvius, 2001). It has been estimated that the size of lipid raft is around 10–200 nm (Pralle et al., 2000) in a dynamic conformation, since they can combine to form larger raft domains.

Domain properties such as composition, size, and lifetimes have been thoroughly investigated (Levental and Veatch, 2016; Sezgin et al., 2017; Levental et al., 2020). The distribution of lipid rafts in cell membranes can vary greatly, from small isolated domains to larger coalescing rafts, depending on a variety of factors, including cell type, specific condition, and type of membrane (e.g., plasma membrane or intracellular membrane). Thus, lipids rafts can be considered like nanodomains enriched in the plasma membrane that can coalesce, forming microdomains platforms for proper cell functioning.

The advancement of technology made it possible to exploit some crucial characteristics of lipid rafts. In fact, since lipid rafts are relatively resistant to non-ionic detergents, such as Triton X-100 (Brown and London, 1998, Raggi et al., 2019), and they are present in low-density fractions after density centrifugation, many authors refer to lipid rafts also as glycolipid enriched and insoluble or detergent-resistant membrane complexes (DRMs) (Simons and Ikonen, 1997).

These characteristics of lipid domains are mainly related to their cholesterol content. In fact, it has been shown that cholesterol sequestering agents selectively destroy rafts. Thus, the use of cholesterol sequestering molecules is a useful tool for identifying proteins as components of the lipid raft or simply copurified contaminants (Foster, 2009), as well as for determining the role of rafts in modulating cellular processes (Mattei et al., 2015; Martellucci et al., 2019).

Noteworthy, these lipid domains show a peculiar fluidity, which allow lateral assembly and rapid reorganization upon diverse biological stimuli. Some molecules associate/dissociate from rafts in a regulated manner depending on their state of activation. These clusters allow the formation of highly efficient lipid–protein molecular associations that operate in several important cellular processes, including membrane trafficking, cell signaling, cell migration, and axonal guidance (Lingwood and Simons, 2010; Sezgin et al., 2017). This structure can concentrate membrane-associated proteins as receptors and molecules involved in signaling pathways (Levental et al., 2010; Martellucci et al., 2018; Mattei et al., 2020; Riitano et al., 2020). Of interest, in polarized cells, lipid rafts show a characteristic sorting on apical surface able to segregate distinct functional proteins, whereas in non-polarized cells, they are distributed randomly on the cell surface.

Lipid rafts play important roles in innate and adaptive immunity; in T lymphocytes, rafts are enriched in many receptors and signaling molecules and participate in T-cell receptor (TCR) triggering and T-cell activation (Varshney et al., 2016; Robinson et al., 2017; Nakayama et al., 2018).

Thus, lipid rafts are thought to function as platforms that recruit specific proteins or concentrate some specific components and exclude others (Wang and Silvius, 2001; Pizzo and Viola, 2004; Pizzo et al., 2004), thus initiating and controlling cell signaling (Simons and Ikonen, 1997; Barbat et al., 2007). Lipid rafts have been proposed to mediate multiple stages of apoptosis (Sorice et al., 2012), including the recruitment of the different key molecules involved in the process, including Fas and the tumor necrosis factor receptor (TNF-α-R) (Garcia-Ruiz et al., 2002; Legler et al., 2003), as well as protein recruitment of the Bcl-2 proapoptotic family, including truncated Bid, t-Bid, and Bax, following the trigger of Fas (Scheel-Toellner et al., 2002).

Lipid rafts are not merely confined to the plasma membrane. In fact, as reported by numerous studies, lipid microdomains are formed similarly in the subcellular organelles, such as Golgi, ER, or mitochondria, termed as *raft-like microdomains* (Garofalo et al., 2005). In particular, functional studies suggest that mitochondrial lipid microdomains participate in the mitochondrial network of fusion and fission during remodeling, as well as in the regulation of cell fate, i.e., survival or death through activation of intracellular signaling (Ciarlo et al., 2010, 2018; Matarrese et al., 2014; Garofalo et al., 2018). Interesting emerging data establish that the interaction of the ER with the mitochondria occurs through endoplasmic reticulum (ER)–mitochondria-associated membrane (MAM) subdomains, and this interaction allows the membrane scrambling, contributing to the multiple functions of ER (Raturi and Simmen, 2013). Since some components of lipid microdomains are present within MAM subdomains (Sano et al., 2009; Garofalo et al., 2016), several authors assume a key role of these subdomains in regulating and influencing a variety of cellular activities (Annunziata et al., 2018), including the early stages of autophagosome formation in mammalian cells (Hamasaki et al., 2013; Garofalo et al., 2016). They are also enriched in caveolin-1 (Sala-Vila et al., 2016), lipid synthesis enzymes (Vance, 1990; Vance et al., 1997), and cholesterol (Area-Gomez et al., 2012; Fujimoto et al., 2012). This particularity suggests that these areas act as non-vesicular lipid transfer sites between ER and mitochondria. In recent years, it has become evident that a complex network of lipid–lipid and lipid–protein interactions contributes to protein sorting and intracellular transport. The hypothesis that the Golgi system sorts the proteins and sends them to the plasma membrane through preferential membrane sites such as lipid rafts, dates back to 1988 (van Meer and Simons, 1988). Moreover, host lipid rafts have been reported to be critically involved in apical targeting, assembly, and virus budding. In this case, the subcellular distribution of lipid raft on internal membranes, including the Golgi apparatus or the ER, has a significant impact in the sorting of proteins and in the trafficking and overall exocytosis of viral proteins, which constitute fundamental steps to support viral infection (Takeda et al., 2003; Von Blume and Hausser, 2019; Stalder and Gershlick, 2020).

Furthermore, at the cellular level, rafts and related membrane microdomains, such as caveolae, characterized by a high expression of caveolin-1, have been proposed to play important roles in the sorting of membrane and non-membrane molecules (Browne and London, 2000; Parton and Richards, 2003). In fact, the study of caveolar platform has been proposed as a potential target to inhibit the entry of SARS-CoV-2 (Filippini and D'Alessio, 2020).

Functionally, lipid rafts host exo-/endocytosis molecular apparatuses that form the functional communication platforms inside and outside the cell (Manes et al., 2003). Thermodynamically, it would be energetically challenging due to the stiff and efficiently packed nature of lipid rafts owing to the fact that fusion mechanism involves processes like membrane bending and non-bilayer lipid intermediates, requiring substantial flexibility of membrane structures (Dadhich and Kapoor, 2020). Thus, Yang et al. (2015) proposed the role of the edges of raft domains, rather than the bulk region, as the preferred sites for fusion. Later on, they verified the mechanisms of fusion to be driven by the effect of hydrophobic mismatch at the edges of raft and not raft (liquid ordered–liquid disordered) domains. Although we cannot refer to the lipid raft as an area dedicated to endocytosis, however, many endocytic (and exocytic) mechanisms involve the lipid rafts to some extent (Pelkmans and Helenius, 2002). Many viruses, including SARS-Cov-2, can enter into the host cells by receptor-dependent endocytosis. One of the best characterized pathways is the clathrin-dependent one, based on viral entry and translocation into endosomes where they are degraded or recycled (Wang et al., 2019). Alternatively, a caveolae-dependent pathway may be used. Caveolae are small invaginations of the plasma membrane that are composed of cholesterol, glycosphingolipids, and caveolin (Filippini and D'Alessio, 2020). Caveolin is able to oligomerize, leading to the formation of caveolin-rich microdomains in the plasma membrane, and subsequently, the caveolar vesicles may fuse with the early endosomal compartment. For instance, coronavirus infection may employ distinct endocytic pathways in the upper and lower respiratory tract related to different signaling molecules. Indeed, a large GTPase, dynamin, which is required for endocytosis, is abundant in the nasal epithelium but undetectable in pneumocytes (Glebov, 2020).

Lipid rafts have been shown to be exploited by intracellular pathogens at different times of the infectious process, as a gateway to the cell. Indeed, many steps of pathogen interaction with host cells, and sometimes all steps within the entire lifecycle of various pathogens, rely on host lipid rafts (Bukrinsky et al., 2020). In addition, the activation of the innate and acquired immune responses by the hosts is regulated by the rafts in many crucial steps; in this regard, some pathogens have the ability to shut down the immunological response by altering the cholesterol content of the lipid raft (immune evasion) (Mackenzie and Khromykh, 2007 and Sen et al., 2011). Possibly, a similar strategy could be shared by SARS-CoV2.

## Role of Lipid Rafts in the Process of Coronavirus Entry Into the Cells

**Figure 1:**
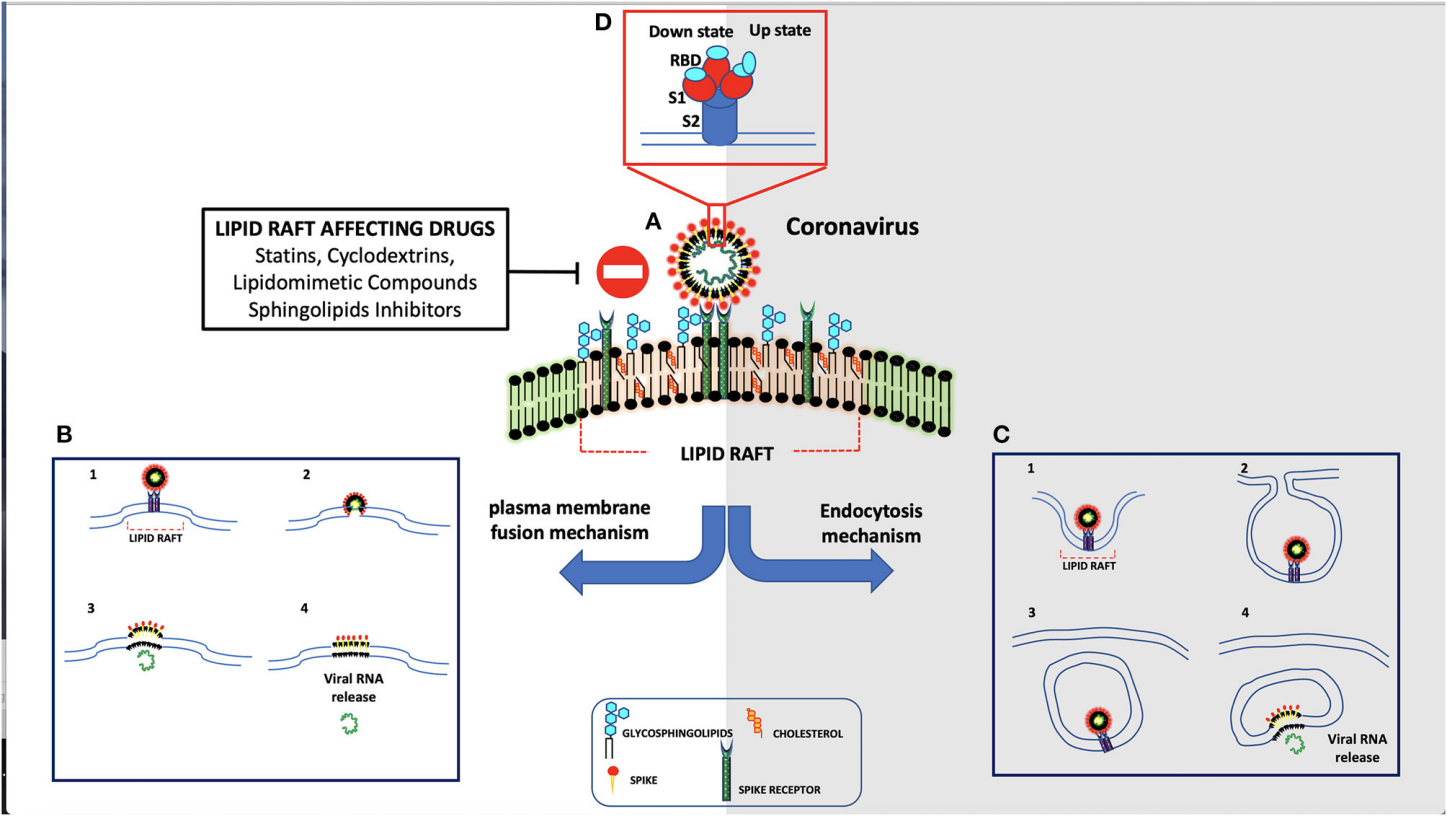
Schematic representation of coronavirus entry mechanism. **(A)** Spike protein interacts through its receptor-binding domain (RBD) with ACE-2 receptor. **(B)** As result of their interaction, spike is activated by human proteases and is internalized by direct fusion with plasma membrane. **(C)** In the absence of proteases, the virus is endocytosed.

Several studies pointed out the key role of lipid rafts during viral infection. Indeed, lipid rafts are involved in different stages of the life cycle of different viruses, including dengue and hepatitis C viruses (Aizaki et al., 2004). Moreover, lipid rafts contribute to the binding and entry of several viruses to host cells, such as human immunodeficiency virus (HIV) (Viard et al., 2002), human herpes virus 6 (Huang et al., 2006), poliovirus (Danthi and Chow, 2004), West Nile virus (Medigeshi et al., 2008), foot-and-mouth disease virus (Martin-Acebes et al., 2007), and simian virus 40 (Parton and Lindsay, 1999). Coronaviruses also interact with lipid rafts for cellular entry (Nomura et al., 2004; Choi et al., 2005; Liao et al., 2006; Li et al., 2007; Pratelli and Colao, 2015; Hu et al., 2016) (Figure 1). The functional role of lipid rafts in this process was supported by the observation that cholesterol depletion prevented coronavirus entry into host cells (Thorp and Gallagher, 2004). Lu et al. reported that lipid rafts are crucial for SARS-CoV entry into cells (Lu et al., 2008). Virus envelope contains the major attachment spike protein (S), the membrane protein (M), and the minor envelope protein (E). Spikes are composed of S protein trimers, which are involved in viral attachment, as well as in the subsequent fusion of viral with cellular membranes (Yang et al., 2012). The S protein comprises two subunits: S1 and S2. Subsequently, the S protein is cleaved by receptor transmembrane serine protease 2 (TMPRSS2) (Hoffmann et al., 2020), a predominantly raft-resident protein (Ballout et al., 2020), with the help of FURIN precleavage, which facilitates the entry of the virus into the cell after binding (Tay et al., 2020). Furin has been found in small fraction on the cell surface, while the predominant amount is in Golgi network (Coutard et al., 2020). Once spike activation has been promoted, virus enters host cells through specific interactions involving cellular surface receptors and viral structural proteins, the viral interactome.

The main receptor for SARS-CoV-2 is represented by the angiotensin-converting enzyme-2 (ACE-2) (Mathewson et al., 2008), a type I transmembrane metallocarboxypeptidase, with its enzymatically active domain exposed on the cell surface. The binding with ACE-2 receptor may facilitate virus surface S1 subunit proteolysis by plasma-membrane-bound serine protease TMPRSS2 and Cathepsine L (CatL), which may be associated with caveolae (Gopal et al., 2006). Once the SARS-CoV-2 reaches intracellular endosomes, CatL becomes the major protease that cleaves the virus S1 subunit (Liu C. et al., 2020). ACE-2 is present on non-immune cells, including endothelial cells, respiratory and intestinal epithelial cells, kidney cells, cerebral neurons, and alveolar monocytes/macrophages. In particular, the ability of SARS-CoV-2 to infect human cells seems to depend by its interaction with human ACE-2 by gln493 residue. However, the S protein uses not only the ACE-2 receptor for entry but also sialic acids linked to host cell surface gangliosides. Indeed, a new type of ganglioside-binding domain within the N-terminal domain of the SARS-CoV-2 S protein has been identified. This domain (111–158), which is a highly conserved sequence, may be responsible for attachment of the virus to lipid rafts, thus facilitating contact with the ACE-2 receptor (Fantini et al., 2020). In particular, ACE-2 is largely colocalized both with raft markers GM1 and caveolin-1. Coronaviruses may enter the host cells either by direct membrane fusion with the plasma membrane or by receptor-mediated endocytosis (Manes et al., 2003). In both processes, lipid rafts play a key role, since they concentrate components of the membrane docking and fusion machinery for endocytosis, such as actin polymerization, which is important for the membrane fusion and endocytosis. When these proteins are concentrated within lipid rafts, their intermolecular interactions are highly facilitated (Nicolau et al., 2006), since partitioning of protein into lipid rafts increases specific interprotein collision rates. Thus, lipid rafts may represent plasma membrane “chambers,” able to increase protein interactions on the plasma membrane and, in turn, increase the collision rate and consequently the efficiency of membrane reactions. In particular, lipid rafts may provide suitable platforms able to concentrate ACE-2 receptor on host cell membranes where they may interact with the S protein on viral envelope. Only in the “open” S conformation, RBD engages PD of ACE-2, and the complex may involve a dimeric ACE-2 that accommodates two S protein trimers (Yan et al., 2020). A clustering of ACE-2 in certain areas of the membrane may allow multivalent binding of virus particles to the cell surface. In this way, microdomains may increase the efficiency of infection but are not an absolute requirement for the entry process. This explanation is in agreement with the finding that cholesterol depletion reduces the susceptibility to infection but does not abolish it (Glende et al., 2008).

In addition, methyl-β-cyclodextrin (MβCD) and mevastatin-induced disruption of lipid rafts inhibited infectious bronchitis virus infection, suggesting that lipid rafts are involved in viral attachment (Guo et al., 2017; Wang et al., 2019). These results indicated that lipid rafts on cell plasma membrane may mediate viral adhesion to facilitate virus endocytosis. It is likely that pathogen–host interactions promote lipid raft clustering and focal adhesion formation during endocytosis.

Thus, we can conclude that lipid rafts may represent attachment factors during the early stages of coronavirus infection.

## Effect of Lipid Rafts-Affecting Drugs on Coronavirus Infection

Lipid rafts affecting drugs, alone or in combination with other compounds, may play a role in antiviral activity. Indeed, as reported above, lipid rafts are crucial components of the viral envelope (Scheiffele et al., 1999), where cholesterol is a known critical structural component. Barman and Nayak (2007) demonstrated that lipid rafts disruption by MβCD-mediated cholesterol depletion is able to reduce influenza virus infectivity. Indeed, it leads to reduced infectivity of virus particles, holes on the viral envelope with consequent effects on particle structure, and altered release of viral proteins. In addition, depletion of cholesterol on host plasma membrane makes it less vulnerable to influenza virus infection. Several authors reported the importance of cholesterol for viral entry into host cells and suggested a role for cholesterol-lowering therapies in reducing SARS-CoV-2 infectivity (Bailly and Vergoten, 2020; Fecchi et al., 2020; Radenkovic et al., 2020; Tang et al., 2020). Drugs such as lovastatin or squalestatin induce cholesterol depletion by inhibiting biosynthesis; as a result, different steps of the virus life cycle can be disrupted. Other drugs, such as filipin, digitonin, nystatin, saponin, or MβCD, cause disruption of lipid rafts in a short period of time, directly removing cholesterol (Barman and Nayak, 2007), although their effects are different at the level of the membrane bilayer (Awasthi-Kalia et al., 2001). For instance, filipin leads to the dispersion of glycosylphosphatidylinositol (GPI)-anchored proteins at the cell surface favoring their release from lipid rafts and decreases the number of caveolae (Robinson and Karnovsky, 1980). Important factors involved in virus infectivity could be afflicted by statins, and some of them are able to reduce the levels of proinflammatory molecules, such as interleukin (IL)-6 and tumor necrosis factor alpha (TNF-α, affecting the autophagic process involved in both viral replication and clearance (Mehrbod et al., 2014). On the basis of these findings, the possibility to undertake studies on patients with severe SARS-CoV-2 infection has been suggested (Fedson et al., 2020). Although there are still controversial theories about the benefits of using statins in patients with SARS-CoV-2, large-scale observational or randomized studies supported this hypothesis (Shu, 2015; Rodrigues-Diez et al., 2020; Subir et al., 2020).

Differently, cyclodextrins have always been considered as excipients with stabilizing and solubilizing properties. At the end of the twentieth century, cyclodextrins have been used as medicinal compounds. The first isolation of cyclodextrins was made by Antoine Villiers in 1981 from starch. Typical cyclodextrins contain three common ring types: (α-CD) alpha-cyclodextrin, (β-CD) beta-cyclodextrin, and (γ-CD) gamma-cyclodextrin. Cyclodextrins can assemble into complexes with various drugs to form host–guest inclusions and have therefore been accepted as pharmaceutical excipients. Cyclodextrins were found attractive for a variety of applications because they could protect sensitive organic guest molecules from oxidation and from volatilization and could make more soluble apolar guests, too. The synthetic derivatives of native cyclodextrins are divided into three groups: ionizable, such as sulfobutylether β-CD (SBE-β-CD); hydrophobic, such as 2,6-di-O-ethyl-β-CD; and hydrophilic, such as 2-hydroxypropyl-β-CD (HP-β-CD). Modified beta-cyclodextrin owns antiviral activities (Braga, 2019). For example, biocompatible sulfonated MβCD mimics some features of heparan sulfate (Jones et al., 2020); in fact, it can act as a broad-spectrum antiviral agent, since it has been proven to reduce influenza A and coronavirus infectivity through depletion of cholesterol. Moreover, drug delivery systems of cyclodextrins can overcome formulation challenges of antiviral drugs improving solubility and bioavailability (Jones et al., 2020).

However, the use of cyclodextrins or statins as active drugs against coronaviruses has some limitations. Indeed, they have a pleiotropic effect in cultured cells by affecting many different signaling pathways. Moreover, in addition to cholesterol, MβCD also extracts other lipids, such as fatty acids and ceramides from cell membranes. Finally, MβCD depolymerizes the actin meshwork, drastically affecting whole cellular architecture.

Antiviral drugs targeting Ebola and HIV have demonstrated encouraging results in SARS-CoV-2 patients, and cyclodextrin seems to be the best excipient to enhance the properties of these drugs, including the antiviral drug Kaletra, a combination of lopinavir and ritonavir, a protease inhibitor for HIV, which demonstrates a benefit in treatment of viral pneumonia (Lim et al., 2020; Wan et al., 2020); the anti-HIV combination lopinavir–ritonavir, currently employed in clinical trials (ClinicalTrials.gov, 2020); and the purine nucleoside Favipiravir, which has recently been authorized for a clinical trial (Liu T. et al., 2020). An additional strategy to disrupt lipid rafts is to use lipidomimetic antiviral agents that alter either viral or host cell membrane blocking viral infection (Nieto-Garai et al., 2018). Therefore, a new antiviral strategy could be assumed based on a rafts-like lipid scaffold.

Remdesivir, an antiviral developed by Gilead Sciences Inc. and previously approved on patients with Ebola, has shown promising results in animal models for MERS and SARS. A formulation with cyclodextrin and remdesivir has been recently proposed (de Wit et al., 2020). In addition, a combination of chloroquine and remdesivir was found to effectively inhibit SARS-CoV-2 *in vitro*. Chloroquine phosphate is an old antimalarial drug and has been effective in inhibiting the exacerbation of SARS-COV-2 pneumonia (Gao et al., 2020). Furthermore, chloroquine displays an immunomodulatory effect by inhibiting TNF-α and IL-6. It also exhibits autophagy inhibitory properties by the elevation of endosomal pH, which may interfere with viral infection and replication (Golden et al., 2015). Hydroxychloroquine presents a terminal hydroxyl group in molecular structure, and several studies have shown the capacity of chloroquine and hydroxychloroquine to bind the sialic acids and gangliosides of the host cells lipid rafts, destabilizing the order, that SARS-COV-2 uses to enter besides the receptor ACE-2 (Fantini et al., 2020; Yuan et al., 2020). This fact suggested a possible additional role for cyclodextrins. Indeed, it was shown that complexation with cyclodextrins lead to an increase in the activity of the antimalarial drug (Torres et al., 2019).

In addition, losartan, a generic blood pressure medication able to block ACE-2 receptor, could be associated with cyclodextrins. Other drugs, such as selective estrogen receptor modulators (SERMs), offer alternative candidate drugs for SARS-CoV-2 (Zhou et al., 2020). Indeed, an overexpression of estrogen receptor, which is localized within lipid rafts (Marin et al., 2012), has been proven to interfere in viral replication through the non-classical pathways associated with estrogen receptor (Lasso et al., 2019). A reasonable solubility is essential to induce bioavailability, and in the case of parenteral therapy, where intravenous solutions must be buffered to physiological pH and be particulate-free, drug solubility is critical, and cyclodextrin represents the best candidate to improve complex therapies. Although researchers are searching for preventive intervention strategies, including interferon therapies, peptides, vaccines, small-molecule drugs, and monoclonal antibodies to treat SARS-CoV-2 infection, these may require several months to test, and all depends on the results of the clinical trials (Shanmugaraj et al., 2020). A few companies are developing actions to accelerate the formation of their neutralizing antibodies, driven by previous successes in the treatment of other diseases (Jiang et al., 2020; Wang C. et al., 2020). Adequate excipients are crucial during shipment and storage to maintain antibody and drugs stability. The protective properties of cyclodextrin, such as the inhibition of proteins aggregation under various stress conditions, have been shown by many case studies (Serno et al., 2010). Finally, researchers have been racing to find possible vaccines for future prevention. Cyclodextrin, as an adjuvant, stabilizes therapeutic monoclonal antibodies, preserves longer immune response, increases antigen (vaccine)-specific antibody titers, and induces type 2 T-helper (Th2) cell response (Onishi et al., 2015) by affecting key signal transduction pathway(s) triggered by lipid rafts.

Further applications for the use of lipid raft affecting drugs are derived from the observations of Zhou and Simmons (2012), who pointed out novel broad-spectrum antiviral compounds to target different stages of the viral life cycle. Certain molecules prove able to be able interfere with the infectivity of some coronaviruses, possibly by viral lipid-dependent attachment to cells (Baglivo et al., 2020). The main pharmacological approaches against coronaviruses are summarized in Table 1.

**Table 1:** Severe acute respiratory syndrome coronavirus-2 (SARS-CoV-2) clinical trials.

**Compounds**	**Action Method**	**References**
Convalescent Plasma	Isolation of IgG and IgM vs. SARS-CoV-2 in order to scale up polyclonal antibody manufacturing to produce treatment cocktails directed against the betacoronavirus causing COVID-19	Shen et al., 2020
Chloroquine/Hydroxychloroquine	Interfering with the glycosylation of angiotensin-converting enzyme 2 (ACE2) and blocking SARS-CoV-2 fusion with the host cell. Impaired terminal glycosylation of ACE2 may reduce the binding efficiency between ACE2 on host cells and the SARS-CoV-2 spike protein	Golden et al., 2015; Torres et al., 2019; Gao et al., 2020
Favipiravir	A guanine analog that inhibit the RNA-dependent RNA polymerase of RNA virus. It has been approved for some other viruses like Human Immunodeficiency Virus (HIV), Hepatitis B Virus (HBV), Hepatitis C Virus (HCV) and influenza	Li and De Clercq, 2020; Liu T. et al., 2020
Remdesivir	A monophosphoramidate prodrug of an adenosine analog with a chemical structure similar to that of tenofovir alafenamide, an approved HIV reverse transcriptase inhibitor. Remdesivir has broad-spectrum activities against RNA viruses such as MERS and SARS in cell cultures and animal models and has been tested in a clinical trial for Ebola	de Wit et al., 2020; Li and De Clercq, 2020
Galidesivir	An adenosine analog that was originally developed for HCV, is currently in early-stage clinical studies evaluating its safety in healthy subjects and its efficacy against yellow fever, and has shown antiviral activities in preclinical studies against many RNA viruses, including SARS and MERS2	Li and De Clercq, 2020
Ribavirin	A guanine derivative approved for treating HCV and respiratory syncytial virus (RSV) that has been evaluated in patients with SARS and MERS, but its side effects such as anemia may be severe at high doses and whether it offers sufficient potency against 2019-nCoV is uncertain	ClinicalTrials.gov, 2020; Li and De Clercq, 2020
human mAb 47D11	A human monoclonal antibody that neutralizes SARS-CoV-2 (and SARS-CoV) in cell culture. This cross-neutralizing antibody targets a communal epitope on these viruses and may offer potential for prevention and treatment of COVID-19	Jiang et al., 2020; Wang C. et al., 2020
Cyclodextrins	The cyclodextrin structure can be modified and used for containment of infections or as virucidal agents. The use of a mouth rinses and/or nasal applications that contain cyclodextrins combined with other drugs could provide a valuable adjunct treatment. Both are locally administered delivery systems that could lower the SARS-CoV-2 viral load	Serno et al., 2010; Lembo et al., 2018; Torres et al., 2019

## Conclusion

It is conceivable that the first contact between coronavirus and host cells occurs into lipid rafts, specialized regions of cell plasma membrane, which provide a suitable platform able of concentrating ACE-2 receptor, thus representing a port of cell entry for viruses.

This review is focused on targeting lipid rafts as a strategy against coronavirus. We report that agents, such as statins or cyclodextrins, can deplete cholesterol and cause disruption of lipid rafts, consequently affecting coronavirus adhesion and binding. Furthermore, these compounds can assemble into complexes with various drugs to form host–guest inclusions and may be used as pharmaceutical excipients of antiviral drugs, such as lopinavir and remdesivir, by improving bioavailability and solubility. Thus, the possible use of drugs affecting lipid rafts in the process of coronavirus entry into the cells introduces a potential new task in the pharmacological strategy against coronavirus.

## Author Contributions

MS, RM, GR, VMan, SM, AL, TG, and VMat wrote the manuscript. All authors contributed to the article and approved the submitted version.

## Conflict of Interest

The authors declare that the research was conducted in the absence of any commercial or financial relationships that could be construed as a potential conflict of interest.
